# A Literature Review of the Lubricants Used in Dermatome-Assisted Split-Thickness Skin Graft Harvest

**DOI:** 10.3390/jcm14124336

**Published:** 2025-06-18

**Authors:** William Wright, Marc Ingram, Quentin Frew

**Affiliations:** 1 St Andrew’s Centre of Plastic Surgery and Burns, Broomfield Hospital, Chelmsford CM1 7ET, UK; q.frew@nhs.net; 2 Ingram Tribology Ltd., Unit 1A Glien Road, Cillefwr Industrial Estate, Carmarthen SA31 3RU, UK

**Keywords:** lubrication, dermatome, MEEK grafting, skin graft, split thickness

## Abstract

**Background:** Split-thickness skin grafts (STSGs) are utilised to close wounds which cannot be closed by primary closure. Dermatome-assisted STSG harvest utilises a lubricant to control friction, which facilitates graft harvest. Many different lubricants are used during graft harvest, although little research has been conducted to identify the optimal lubricant. Furthermore, new techniques such as Meek grafting are incompatible with commonly used oil-based lubricants. **Method:** A literature search was conducted, following the PRISMA protocol. 173 records were screened with 6 included in this study. We also reviewed the literature on lubricants in other biotribological systems including shaving. **Results:** We found support for numerous lubricants, including: mineral oil, catheter gel, chlorhexidine, saline and ultrasound gel. Evidence consisted of expert opinions, and one blinded comparative review. There was no consensus on the optimal lubricant, and we did not find evidence that lubricant compatibility with Meek grafting had been assessed. **Conclusions:** Presently, lubrication choice in STSG harvest lacks a scientific basis, and further research is needed to design a bespoke, Meek-compatible lubricant which considers only four of Engelhardt’s characteristics (1. cost-effectiveness; 4. lubrication; 6. no side effects; 8. practicability) to be essential. This should be followed by a blinded trial of lubricants.

## 1. Introduction

Split-thickness skin grafts (STSGs) are essential tools in reconstructive surgery and are frequently utilised to close wounds which cannot be closed by primary closure. The expansion enabled by meshed STSGs allows for even larger wounds to be successfully grafted, which is particularly important in burn reconstruction. STSGs offer benefits over secondary intention healing including reduced time to healing, improved functional outcome and preferable long-term scar outcome. Cubison et al. demonstrated a low risk of hypertrophic scar formation when a wound heals within 21 days [1], which STSGs can facilitate. Historically, STSGs were harvested using a Watson or Humby knife. However, with recent technological advancements, the gold standard for modern STSG harvest is a power-driven mechanical dermatome, which has increased the precision and consistency of graft harvest. A mechanical dermatome consists of an oscillating cutting blade in between a metal plate; it enables the uniform harvest of a thin layer of skin consisting of the epidermis and a portion of the dermis from the patient. Dermal preservation is directly linked to wound healing and scar outcome, and therefore the consistent depth of graft harvest allows for predictable wound healing at both the patient’s graft and donor site.

The operator of a dermatome can significantly affect the quality of the harvested graft by changing two variables: the angle between the blade and the skin and the pressure applied. The careful control of these two variables throughout harvest will improve the quality of the STSG. One challenge for the operator is that the friction coefficient of skin is highly variable across the surface of the human body and is influenced by a number of intrinsic factors including the location on the body and extrinsic factors including temperature and hydration status [2,3,4].

The application of a lubricating agent to the dermatome and donor site can maximise the ease of harvesting a uniformed graft [5,6] by enabling the operator to keep the two abovementioned variables as constant as possible. Lubrication allows for this by causing an absolute reduction in the friction between the dermatome and skin, as well as causing a reduction in the variation in the coefficient of friction across the area of skin from which the graft will be harvested (the donor site). Optimal lubrication will help to minimise skipping (cutting too shallow) and gouging (cutting too deep) at the donor site and thus allows for the harvest of a graft with a constant thickness of the dermis. Consequently, improper lubrication may be contributing to the number of failed skin grafts, extending healing times and increasing discomfort for the patient. For example, Nomani et al. demonstrated an improved graft take when mineral oil was used as a lubricant (83% graft take at 1 week) compared to talcum powder (68% graft take at 1 week), which was being used as a lubricant at the time [7].

The re-emergence of Meek grafting is posing a new challenge to surgeons when selecting a lubricant in dermatome-assisted STSG harvest because oil-based lubricants are contraindicated in Meek grafting due to their interference with the adhesive glue used in Meek. The effects of lubricant choice on other specialist grafting techniques such as Recell have not been assessed.

Biotribology is the study of friction on biological surfaces and provides us with a framework for understanding the interaction between surgical instruments and human tissue. Biotribology is frequently encountered in orthopaedic surgery with the design of prosthetic joints; however, its role in plastic and dermatological surgery is embryonic. Despite the significance of lubrication in STSG harvest, only a very limited amount of research has been conducted into the matter. However, a greater amount of research has been conducted into other areas of dermatological biotribology with relevant parallels. This includes designing the optimal razor blade and shaving cream to control friction during shaving when using a razor on human skin.

Given the lack of research into lubricants in STSG harvest, there is no formal consensus on which lubricant is optimal. It is likely that many different lubricants are being utilised in current practice, with selection being influenced by a surgeon’s or healthcare provider’s preference and availability. In the experience of the authors, mineral oil is the most commonly used lubricant.

We therefore conducted a review to compile and appraise current research into the choice of lubricant in STSG harvest and to identify desirable characteristics associated with an optimal STSG lubricant. We went on to suggest areas for further research to address the paucity of literature on the matter and used this as a stepping stone for the development of an evidence-based lubricant for dermatome-assisted STSG harvest.

## 2. Methods

This study used the PRISMA [8] (Preferred Reporting Items for Systematic Reviews and Meta-Analyses) protocol to conduct a systematic literature search. The PICOT [9] format was used to structure the question for this review: “In patients undergoing a dermatome-assisted split-thickness skin graft harvest (population), which lubricant (intervention and comparator) is associated with optimal outcomes including graft take, user satisfaction and adverse outcomes (outcomes)?” The secondary goal was to identify the essential characteristics a lubricant must possess in order to be optimal for STSG harvest.

**Figure 1:**
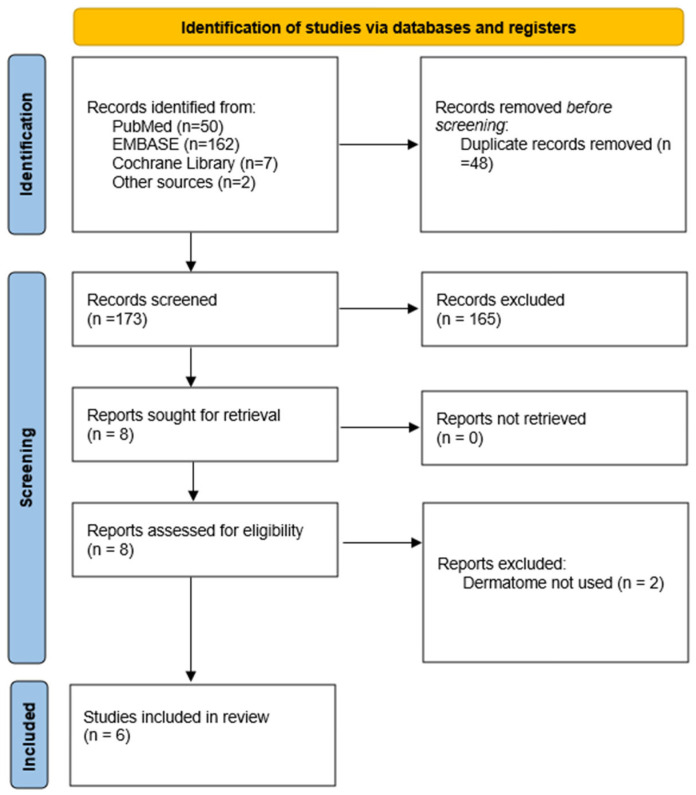
PRISMA search strategy results. Template source: Page M.J. et al. [8].

PubMed, EMBASE and Cochrane Library were utilised as the search system with the following search terms: “(Skin) and (graft*) and (lubrica*)”. The use of wildcards and Boolean operators expanded our search results, and we did not exclude any search result based on the age of the study. The references of selected studies were manually searched for articles that were not located during the database searches. There was only one exclusion criterion applied to the search results, which removed studies which did not use a dermatome to harvest the STSG. Our results included blinded controlled trials, original research studies and expert opinions. Figure 1 shows the “PRISMA protocol” flowchart for our literature review, detailing the number of studies identified and the final number included.

### Findings (See Table A1 for Results Table)

The results from our literature search are detailed in Table A1. Firstly, we will review the characteristics of a lubricant that have been suggested by others to be essential. Then we shall present the lubricants which have been studied with reference to the characteristics identified.

Engelhardt et al. [10] identified nine characteristics of an optimal lubricant for STSG, which are listed in Table 1. Throughout this article we will refer to these characteristics by their number. In our literature search, we did not find any alterative characteristics, nor did we identify any suggested refinements to Engelhardt’s list.

This literature review identified the use of a wide selection of water- and oil-based lubricants ranging from off-the-shelf solutions, including mineral oil, to repurposed, readily available substances (for example, ultrasound lubricant), to more creative, purpose-made solutions which are produced by the end user at the time of use.

Engelhardt et al. report using catheter gel containing lidocaine (local anaesthetic) and chlorhexidine (antiseptic) with great success as a lubricant in STSG harvest. They believe it has every characteristic they identified to be important except for enabling an enhancement in wound healing. They believe it is superior to mineral oil (which they report lacks characteristics 2, 3, 7) and saline (which only has characteristics 1, 5, 6, 8).

Sams et al. [11] offered an expert opinion that mineral oil “facilitates smooth movement” of the dermatome; however they did not make a comparison between lubricants.

Rodrigues et al. [12] affirmed the drawbacks of paraffin, which include “persistent greasiness, preventing the use of adherent dressings”, and the failure of saline to provide sufficient lubrication. They advocated a weak dilution of chlorhexidine soap, such as HiBiSCRUB diluted 1 part in 200 parts saline. This solution is water-soluble, unlike paraffin, and is more lubricating than saline. However, they did not directly compare the effectiveness of the lubrication provided by paraffin and diluted chlorhexidine soap.

Wettstein et al. [13], despairing at the drawbacks of paraffin (including the poor adherence of the dressing), found a 100% success rate in graft harvest without an unwanted termination or perforation of the graft when using saline in 42 STSGs. They concluded that the use of paraffin was not necessary and that saline should be used instead given its advantageous handling properties and compatibility with adhesive dressings.

Braza et al. [14] reported the use of an ultrasound lubricant to be beneficial in the absence of mineral oil following three successful procedures. They believed it had a number of Engelhardt’s characteristics (1, 4, 5, 6, 8; as per Table 1).

Beckett et al.’s blinded, randomised control trial tested 5 lubricants on a porcine skin model. The lubricants were assessed for five characteristics: ease of use, graft uniformity, skipping, gouging and overall satisfaction. The novel water-based lubricant (120 g sterile bacteriostatic water-based surgical lubricant diluted to 200 cc total with sterile water) came out superior and was statistically significantly more satisfactory than glycerin and mineral oil. Given the design of this trial, the evidence is of a higher quality than that of the other studies which are based on an individual’s, or a group of individuals’, clinical experience.

There was insufficient information provided in the studies regarding other variables which should be controlled to aid comparison. None of the studies reported on donor site preparation prior to the application of a lubricant. We could not identify whether the anatomical location of the donor site was controlled for within or between studies. Finally there was no information on whether donor site infiltration was used prior to graft harvest to achieve turgor.

Two studies were formally excluded from this review for using a Humby knife rather than dermatome to harvest the STSG; however, these studies had limited findings on lubricants. Nomani et al. [7] conducted a comparative trial which found a correlation between the use of mineral oil and greater graft take at 1 week compared to the use of talcum powder but did not comment on the lubrication provided by the two agents. Morritt et al. [15] proffered that Jelonet (containing soft white paraffin) was a better means of applying lubricant than liquid mineral oil but did not compare the lubrication properties of the two substances.

## 3. Discussion

Engelhardt identified what he believed to be the nine characteristics that an optimal lubricant should possess. As mentioned, we did not find any disagreement or refinement of Engelhardt’s nine characteristics. In fact, considering the dominance of Engelhardt’s list on our perception of the optimal lubricant, much of the discourse on lubricants used in STSG harvest has been framed in terms of Engelhardt’s characteristics.

It is the authors’ opinion that whilst Engelhardt et al.’s suggested nine characteristics of the optimal lubricant are all beneficial, they are by no means essential to a lubricant for STSG harvest. The fundamental function of the lubricant should be to facilitate the ease and quality of graft harvest by controlling the friction between the skin and the dermatome. The lubricant should not cause side effects and ought to be cost-effective and practical (characteristics 1, 4, 6, 8). However, the lubricant does not necessarily need to participate in the following: pain reduction (characteristic 2); haemostasis (characteristic 3); enhancement of wound healing (characteristic 7) or antiseptic effect (characteristic 9). These are all important aims in a successful STSG harvest; however, they could be better achieved by other agents which are independent of the lubricant. For example, the enhancement of wound healing, through optimal wound care and dressings, and pain reduction can be achieved with oral analgesics and local anaesthetic at the donor site. Alternative agents or techniques may also be more cost-effective at achieving these aims rather than trying to incorporate additional properties into a lubricant such as haemostatic and antiseptic effects. Furthermore, for a lubricant to satisfactorily possess all nine of Engelhart’s characteristics, it may result in a compromise on what we believe to be the essential characteristics (characteristics 1, 4, 6, 8) of the optimal lubricant.

Table A2 presents the findings of this review by highlighting which of Engelhardt’s characteristics each lubricant has been identified to possess. It is apparent that significantly more research is required to assess each lubricant against all nine of Engelhardt’s characteristics. This would then enable an accurate and fair comparison between the agents. Unfortunately, we did not identify comparable studies which looked at the effects of lubrication choice on the following: pain reduction, donor site morbidity, and infection risk.

Of the six studies included in this review, five were expert opinions with limited or no empirical evidence provided, which further hindered this review’s ability to compare lubricants between studies. Beckett et al.’s study was a blinded controlled trial and thus the only study with a more rigorous experimental method. Her team identified a purpose-made water-soluble solution to be statistically significantly better than mineral oil.

It is important to consider the limitations of Beckett et al.’s trial, which they identified as follows: the limited range of lubricants trialled; the substitution of human skin with a porcine model, which is inherently greasier and fails to replicate the effects of human bodily fluids; and the lack of clinical correlation. These are limitations that any future trial should attempt to mitigate as much as possible.

### 3.1. Lubrication

Engelhardt identified lubrication as the fourth characteristic of an optimal lubricant. However, he did not expand on this primary function of an optimal lubricant. We need to identify, as precisely as possible, what optimal lubrication is in the context of dermatome-assisted STSG harvest. Lubrication is the process of reducing friction, and the effectiveness of a lubricant can be quantified using a tribometer. The quantification of the effect that different lubricants have on friction should be an essential component in future research to allow for a fair comparison between lubricants.

The skin graft harvesting process is a manual process involving a complex combination of downward force and forward force. This is controlled by the surgeon. The surgeon will aim to complete an accurate skin graft at a set depth into the dermis, controlling the speed of the dermatome cutter (forward force), the pressure (downward force) and the angle between the skin and dermatome. It is assumed that the friction between the cutter should be controlled and optimised to aid this process. This would involve reducing the variation in the friction between the dermatome and skin across the donor site to ensure a smooth and thus even-thickness graft harvest. To achieve this, the design of the lubrication system should take into account a couple of key points:

- The preference of the surgeon—the surgeon may be familiar with a certain friction characteristic of the lubricant/dermatome/skin system and may dislike a large change to the feel of the force encountered when sliding the dermatome. This is known as haptic feedback.
- The friction may need to be controlled within a set range, as opposed to just reducing it as far as possible to ensure a controlled STSG harvest.

It is possible to design a lubricant which reduces friction between the dermatome and skin to near zero. However, this would not be beneficial, and it would significantly impair the surgeon’s control of the dermatome, resulting in the dermatome frequently slipping and thus causing significant harm to the patient and surgeon. Similarly, excessive friction may cause thermal damage to the skin and increase unwanted incidents of gouging and skipping. High values for the coefficient of friction will increase the force required by the surgeon to be applied during graft harvest, which can lead to surgeon fatigue. This is particularly significant when long lengths of graft are required to be harvested. As such, the optimal lubricant should reduce the friction between the skin and dermatome to a level that preserves haptic feedback without excessively reducing the coefficient of friction to the point that the control of the dermatome is compromised. This “sweet spot” can only be identified by a formal trial which utilises a number of surgeons who each compare a range of lubricants, each of which cause a different degree of reduction in friction between the skin and a dermatome. This trial would need to record subjective metrics, including surgeon satisfaction and ease of use, and objective metrics including the coefficient of friction and the consistency of graft thickness. The effects of a lubricant on cutting dynamics could also be assessed using high-speed imaging.

Despite the limited literature on lubrication in STSG harvest, more research has been conducted into other dermatological biotribological systems that are more common in everyday life such as facial hair shaving. The process of shaving is in many ways similar to taking an STSG, and Van Der Heide et al. point out the three main variables in shaving as the following: the pressure applied by the operator, the sliding velocity and lubrication [16]. Researchers in this field have shown that human skin is characterised by nonlinear viscoelastic material behaviour [3,4] and that the coefficient of friction on skin varies with age, gender, ethnicity and anatomical location on the body [17,18,19]. Separate research by Veijgen et al. shows that the coefficient of friction of skin varies with operational conditions, environmental conditions including humidity, material selection and possibly the type of motion used [20].

During shaving, there are two contributors to overall friction which affect razor glide: adhesion friction between the razor and skin and hair cutting friction. Interestingly, research by Mahdi [21] has shown that the proportion of total friction caused by adhesion friction and cutting friction that contributes to overall friction depends on the type of lubricant used. Further research is required into dermatomes and lubricants to assess the components of total friction during STSG harvest.

Other research from the shaving industry has looked at the impact of hydration on friction. Skin hydration has been shown to affect the coefficient of friction, with drier skin having a lower coefficient of friction compared with hydrated skin. However, it is not a linear relationship as very wet skin has a coefficient of friction similar to that of dry skin [21]. Water was studied as a lubricant by Johnson et al. [2] who showed that wet skin had a higher level of friction compared to dry skin and that water reduces control, owing to sticking and slipping [21]. Many razors currently on the market utilise a water-soluble lubricating strip (typically polyethylene oxide) to reduce drag and enhance shave comfort [22]. Further research into lubricants for STSG harvest should consider the impact of the lubricant on skin hydration. It would be possible to quickly and accurately measure the friction of a model dermatome/skin system using the instruments available in biotribology.

It is also worth noting that it has been recognised within bodies of research on shaving that there is large variation in shaving behaviour among users, including in the speed and force of shaving strokes [23]. The authors expect a similar variation amongst dermatome operators.

### 3.2. Specialist Considerations

A new consideration that has been mentioned but warrants further discussion is the compatibility of lubricants used in STSG harvest with the Meek grafting process. Meek grafting was first described in 1958 by Meek [24], but it was not until the process was refined by Kreis in 1993 that it became more widely adopted [25]. The Meek grafting technique has facilitated a significant advance in the use of STSGs to reconstruct large defects that were previously too big to be grafted in one procedure or when there are limited appropriate donor sites available [26]. The Meek technique involves cutting a harvested STSG into small, regular-sized squares which are then attached on a specialised gauze which allows grafts to expand by up to nine times. Another advantage of the Meek technique is the reduction in wound healing times, which is an important factor in reducing overall morbidity. Burn wounds which have been Meek-grafted have been reported to close in half the time compared to wounds treated with a traditionally meshed STSG [27].

However, oil-based lubricants are contraindicated in the Meek grafting process because of their undesirable interaction with the Meek adhesive which plays a critical role in the process. The adhesive is used to transfer the cut skin onto the specialised Meek expansion gauze and to ensure the pieces of graft remain in the precise position at the recipient site. Oil-based lubricants may interfere with graft adhesion to the gauze, and the failure of this process may impede graft integration and delay reepithelization. At present, saline solution is the most commonly used lubricant in STSG harvest for Meek grafting and is recommended by Meek equipment suppliers. We did not identify any available data regarding Meek and lubricants. Given the valuable role this technique plays in complex skin grafting procedures, further research is required to design an optimal lubricant which is compatible with the Meek grafting process. Unfortunately, the composition of the Meek adhesive used is not available in the public domain, which may hinder research. However, this literature review identified a number of alterative water-based lubricants, including an ultrasound lubricant, catheter gel and HiBiSCRUB solution, which have been used in STSG harvest and all of which are reported to have superior lubrication properties to saline. Their interaction with the Meek adhesive should be assessed.

Another specialist technique which utilises STSGs and enables a significant expansion of up to 80 times between the donor site and recipient site is ReCell [28]. ReCell was developed by Professor Fiona Wood and Marie Stoner in the 1990s. The authors of this review are not aware of any studies looking at the potential effects of the lubricant used to harvest the STSG which is used in ReCell on the wound healing time and percentage graft take. This is a consideration that future research could assess.

## 4. Conclusions

Effective lubrication in dermatome-assisted STSG harvest contributes to graft quality, which goes on to affect wound healing times and the patient’s clinical outcomes. This literature review identified a wide range of oil- and water-based lubricants that are used in STSG harvest, as well as revealing the lack of sufficient evidence to support the use of one lubricant universally. In addition, we identified that there has been limited discourse on what characteristics the optimal lubricant should possess. We refined the list suggested by Engelhardt to the following: cost-effectiveness, lubrication, no side effects and practicability. Furthermore, we discussed how optimal lubrication can be identified, which is important when designing new lubricants or when comparing the lubrication characteristics of different lubricants in future trials.

Given the important role lubrication plays, significantly more research is required. We recommend a national or international survey of clinicians involved in dermatome-assisted STSG harvest to identify the range of lubricants currently used in clinical practice. Meanwhile we should seek the creation of new lubricating agents which are purpose-designed, based on the four refined characteristics taken from Engelhardt’s original nine.

Following this, there is a need for an extensive, randomised, blinded, controlled trial assessing a wide range of lubricants, including new agents. The trial should test lubricants on living human subjects and in different anatomical locations given the range in the value of the coefficient of friction for skin. The measurement of the friction coefficient between the skin and dermatome in situ would require specialist tribological equipment. However, this would allow researchers to quantify the comparison of lubricants. Separately, the uniformity of the harvested graft could be measured and compared. We recommend utilising a number of trained practitioners who can assess lubricants against the four characteristics we identified as essential from Engelhardt et al.’s original nine. Another consideration would be the surgeon’s “perception” of the lubricant system, as we suspect that surgeons may come to prefer their own systems based on experience. It is also important that haptic feedback is preserved and that the control of the dermatome is not compromised. It would also be beneficial to assess patient-related outcome measures in addition to outcomes such as healing times, graft take, donor site morbidity and adverse scarring.

If a trial were to assess lubricants against all nine of Engelhardt’s characteristics, it would need to consider the relative weighting of these nine characteristics. Finally, each lubricant would need to be separately assessed for its compatibility with the Meek grafting technique and other specialist grafting technologies.

## Figures and Tables

**Table 1:** Characteristics of optimal lubricant for split-thickness skin harvest. Engelhardt et al. [10].

Characteristics of Optimal Lubricant
Cost-effectiveness.
2. Pain reduction.
3. Haemostasis.
4. Lubrication.
5. Solubility.
6. No side effects.
7. Enhancement of wound healing.
8. Practicability.
9. Antiseptic effect.

## Appendix A

**Table A1:** Results of literature search showing included studies.

Author	Year	Subject	Research Location	Method	Result	Limitations
Allison R. Beckett et al. [6]	2019	Porcine skin model	Arnold Luterman Regional Burn Center, University of South Alabama Medical Center	Compared five lubricants whilst harvesting split-thickness skin graph (glycerin, mineral oil, saline, poloxamer 188, surgical lube + sterile water (120 g sterile bacteriostatic water-based surgical lubricant diluted to 200 cc total with sterile water)) Results recorded on Likert scale (1–5) Blind comparison of solutions with dry control Four harvesters	Dry control = 1.1 ± 0.1; glycerin = 2.62 ± 1.02; saline = 3.88 ± 0.81; mineral oil = 3.75 ± 1.00; novel water-based lubricant solution = 4.63 ± 0.71; and poloxamer 188 = 3.88 ± 0.81. “glycerin had statistically significantly lower scores that all other solutions (*p* < 0.01)”. “Novel water based surgical lubricant had significantly higher mean scores than glycerin (*p* < 0.01) and mineral oil (*p* < 0.05).” All solutions were superior to dry control. No solution had a statistically higher score than saline. Glycerin was least cost-effective. Surgical lube + sterile water was most cost-effective.	May be skewed due to the greasy nature of butcher shop porcine skin. No “correlation to clinical performance and outcome”.
Timm O. Engelhardt et al. [10]	2012	Patients	Innsbruck Medical University, Austria	Clinical experience Catheter gel on 12 females and 18 males between 2010 and 2011	Identified nine major characteristics of optimal lubricant for split-thickness skin harvest. Drawbacks of paraffin (possible negative influence on wound healing no beneficial effect on haemostasis; no influence on pain reduction). Also hard to apply dressing, and solvents used to remove paraffin may cause skin irritation. Saline meets characteristics 1, 5, 6 and 8. Used catheter gel containing lidocaine hydrochloride and chlorhexidine hydrochloride. No technical complications, good healing. Meets all criteria except 7 (wound healing). Benefits of anaesthetic and antiseptic properties of soluble gel.	
HUNTER H. SAMS et al. [11]	2004	Patients	The Vanderbilt Clinic, Nashville	Clinical experience	Affirmed that use of lubricant (mineral oil) had benefit in skin graft harvest by facilitating movement of dermatome. Also found benefit of tongue depressor to create flat surface and semipermeable membrane to decrease curling and contraction of graft and decreases graft tears and damage and facilitates transfer to recipient wound bed.	
JN Rodrigues et al. [12]	2012	Patients	Nottingham University Hospitals	Clinical experience	Limitations of paraffin (“persistent greasiness, preventing the use of adherent dressings to the donor site”) and saline (“which may note lubricate adequately, affecting graft quality”). “Weak dilution of chlorhexidine soap offers more slippery surface than saline. Water solubility allows it to be washed off.“ Diluted 1 part in 200 to avoid foaming in dermatome.	
Reto Wettstein et al. [13]	2011	42 patients	Lausanne, Switzerland	Clinical experience based on 42 STSGs from 15 women and 22 men	Normal saline showed 100% success rate. Paraffin moderately influences cost of operation but “can render dressing adherence cumbersome and hazardous”.	Assessed in terms of graft take. No comment on improved lubrication/ease of harvest.
Matthew E. Braza et al. [14]	2021	Patients	Michigan	Clinical experience based on 3 procedures	Alternative to mineral oil. Used sterile water-soluble ultrasound lubricant. “Similar to catheter lubricant, is cost-effective, water soluble, without known side effects, practical, and a lubricant”.	

**Table A2:** Evidence for which of Englehardt’s characteristics is possessed by each lubricant.

	Lubricant								
Characteristic	Dry Control	Glycerin	Saline	Mineral Oil/Paraffin	Beckett’s Novel Water-Based Solution	Poloxamer	Catheter Gel	Chlorhexidine Soap	Sterile Water-Soluble Ultrasound Lubricant
**1 Cost-effectiveness**		Beckett: Least cost-effective	Engelhardt: Achieved	Wettstein: More expensive than saline despite 100% graft success using saline	Beckett: Most cost-effective		Engelhardt: Achieved		Braza: Achieved
**2 Pain reduction**				Engelhardt: No influence on pain reduction			Engelhardt: Achieved		
**3 Haemostasis**				Engelhardt: No beneficial effect			Engelhardt: Achieved		
**4 Lubrication**	Beckett	Beckett: Significantly worse than novel water-based solution	Rodrigues: Not achieved Wettstein: 100% success rate using normal saline for graft harvest	Beckett: Significantly worse than novel water-based solution	Beckett: Significantly better than glycerin (*p* < 0.01) and mineral oil (*p* < 0.05)		Engelhardt: Achieved	Rodrigues: More effective than saline	Braza: Achieved
**5 Solubility**			Engelhardt: Achieved				Engelhardt: Achieved	Rodrigues: Achieved	Braza: Achieved
**6 No side effects**			Engelhardt: Achieved	Engelhardt: Solvents used to remove paraffin may cause skin irritation			Engelhardt: Achieved		Braza: Achieved
**7 Enhancement of wound healing**				Engelhardt: Possible negative influence on wound healing			Engelhardt: Not achieved		
**8 Practicability**			Engelhardt: Achieved	Engelhardt: Harder to apply dressings Rodrigues: Prevents use of adherent dressings Wettstein: “Ca render dressing adherence cumbersome and hazardous”.			Engelhardt: Achieved		Braza: Achieved
**9 Antiseptic effect**							Engelhardt: Achieved		
